# Aufbau eines neuen Patientenregisters für Gorlin-Goltz-Syndrom

**DOI:** 10.1515/medgen-2023-2007

**Published:** 2023-04-05

**Authors:** Franziska Schnabel

**Affiliations:** Universitätsklinikum Leipzig Institut für Humangenetik Leipzig Deutschland

Am Institut für Humangenetik des Universitätsklinikums Leipzig wird ein neues Register für Patient*Innen mit Gorlin-Goltz-Syndrom etabliert. In einem Online-Fragebogen sollen die klinischen und genetischen Daten der Betroffenen erfasst werden. Hierbei werden im Detail die Symptomatik der einzelnen Organsysteme, der klinische Verlauf, die bisherigen Therapien sowie die Ergebnisse der durchgeführten genetischen Diagnostik erfragt. In das Register können alle Patient*Innen aufgenommen werden, die die klinischen Kriterien des Gorlin-Goltz-Syndroms erfüllen (siehe Tabelle), wobei insbesondere auch Betroffene mit unklarer genetischer Ursache eingeschlossen werden können. Die Dateneingabe kann von den Patient*Innen selbst oder von ihren behandelnden Ärzt*Innen nach Zustimmung in einer entsprechenden Einwilligungserklärung erfolgen. Um das Patientenregister zukünftig auch international zur Verfügung stellen zu können, ist zudem eine Übersetzung des Online-Fragebogens in weitere Sprachen geplant.

Das Gorlin-Goltz-Syndrom, auch als naevoides Basalzellkarzinomsyndrom bezeichnet, ist typischerweise durch multiple Basalzellkarzinome gekennzeichnet, die sich im mittleren Lebensalter manifestieren. Ein Großteil der Betroffenen weist zudem odontogene Keratozysten im Kieferbereich auf, die aufgrund des destruierenden Wachstums und der hohen Rezidivrate operativ entfernt werden müssen. An den Handflächen und Fußsohlen können punktförmige Grübchen vorliegen, die als palmoplantare Pits bezeichnet werden. Radiologisch ist meist eine Verkalkung der Falx cerebri, seltener auch des Diaphragma sellae und des Tentorium cerebelli nachweisbar. Als weitere Symptome sind Lippen-Kiefer-Gaumenspalten und Skelettanomalien wie fusionierte Rippen und Wirbelkörper, Skoliose sowie prä-/postaxiale Polydaktylie beschrieben. Fazial können eine relative Makrozephalie mit prominenter Stirn, vergröberten Gesichtszüge und Hypertelorismus auffallen. Assoziierte Augenfehlbildungen umfassen Strabismus, kongenitalen Katarakt, orbitale Zysten, Mikrophthalmie und Anomalien des retinalen Pigmentepithels. Zusätzlich können ovarielle und kardiale Fibrome sowie selten auch mesenteriale und pleurale Zysten bestehen. Bei ca. 5 % der Betroffenen tritt die Erkrankung mit einem Medulloblastom in den ersten Lebensjahren in Erscheinung.

Das Krankheitsbild folgt einem autosomal dominanten Erbgang und tritt in ca. 65–80 % der Fälle familiär gehäuft auf. Die Prävalenz wird auf 1:40.000 bis 1:60.000 geschätzt. Bei ca. 70 % der Patienten, die die klinischen Kriterien eines Gorlin-Goltz-Syndroms erfüllen, wird eine ursächliche genetische Veränderung im *PTCH1*-Gen gefunden. Ca. 5 % der Betroffenen weisen eine genetische Veränderung im *SUFU*-Gen auf. Darüber hinaus sind Einzelfälle mit einer Variante im *PTCH2*-Gen beschrieben. Sowohl *PTCH1*, als auch *SUFU* und *PTCH2* sind Bestandteil der Sonic-Hedgehog-Signalkaskade. Allerdings kann bisher bei nicht allen Patient*Innen mit klinischer Diagnose eines Gorlin-Goltz-Syndroms eine molekulare Ursache identifiziert werden.

Die Sonic-Hedgehog-Signalkaskade gilt als wichtiger Signaltransduktionsweg während der embryonalen Neuralrohrentwicklung, wohingegen die Signalkaskade in adulten Geweben zumeist herunterreguliert ist. Eine aberrante Aktivierung beispielsweise durch genetische Veränderungen in den zugehörigen Interaktionspartnern kann zu neoplastischem Zellwachstum und insbesondere zur Bildung von Basalzellkarzinomen führen. Sonic-Hedgehog-Inhibitoren wie Vismodegib und Sonidegib sind zur Behandlung von lokal fortgeschrittenen bzw. metastasierten Basalzellkarzinomen zugelassen und können eine deutliche Reduktion der Tumormasse erzielen.

Mit dem neuen Patientenregister sollen einerseits das klinische Spektrum und die genetischen Ursachen des Gorlin-Goltz-Syndroms besser charakterisiert und somit zu einer Optimierung der Patientenversorgung beigetraten werden. Andererseits soll das Register aber auch die Grundlage für weiterführende Studien zur Identifizierung neuer krankheitsassoziierter Gene und Mechanismen bilden und die Kontaktvermittlung für zukünftige standardisierte Therapiestudien ermöglichen.

Wissenschaftliche Ansprechpartnerin

Dr. med. Franziska Schnabel

Institut für Humangenetik, Universitätsklinikum Leipzig

Tel.: 0341-97 23825

Email: franziska.schnabel@medizin.uni-leipzig.de

Register: https://www.uniklinikum-leipzig.de/einrichtungen/humangenetik/Seiten/de_gorlin.aspx

**Diagnosekriterien:** Die klinische Diagnose eines Gorlin-Goltz-Syndroms kann gestellt werden, wenn mindestens 2 Haupt- und 1 Nebenkriterien bzw. mindestens 1 Haupt- und 3 Nebenkriterien erfüllt sind.

**Hauptkriterien**	**Nebenkriterien**
– Multiple Basalzellkarzinome (> 5) oder eines vor dem 30. Lebensjahr	– Medulloblastom in der Kindheit
– Odontogene Keratozyste des Kiefers	– Mesenteriale/pleurale Zyste
– Verkalkung der Falx cerebri	– Makrozephalie (> 97. Perzentile)
– Palmare/plantare Pits (≥2)	– Lippen-Kiefer-Gaumenspalte
– Verwandter ersten Grades mit Gorlin-Goltz-Syndrom	– Wirbelkörper-/Rippenfehlbildung
	– Prä-/postaxiale Polydaktylie
	– Kardiales/ovarielles Fibrom
	– Augenanomalie

